# Study on the Interaction of β-Cyclodextrin and Berberine Hydrochloride and Its Analytical Application

**DOI:** 10.1371/journal.pone.0095498

**Published:** 2014-05-08

**Authors:** Baoxiu Jia, Yuqin Li, Decai Wang, Rui Duan

**Affiliations:** College of Pharmaceutical, Taishan Medical University, Tai’an, P.R. China; CNR, Italy

## Abstract

The fluorescence enhancement of berberine hydrochloride (BBH) as a result of complex with β-cyclodextrin (β-CD) is investigated. The mechanism of the inclusion was studied and discussed by spectrofluoremetry and infrared spectrograms. The results showed that a 1∶1 (β-CD: BBH) complex was formed with an apparent association constant of 4.23×10^2^ L/mol. Based on the enhancement of the fluorescent intensity of berberine hydrochloride, a new spectrofluorimetric method for the determination of BBH in the presence of β-CD was developed. The linear range was 1.00∼4.00 µg/mL with the detection limit of 5.54 ng/mL. The proposed method was successfully applied to the determination of BBH in tablets.

## Introduction

**Figure 1:**
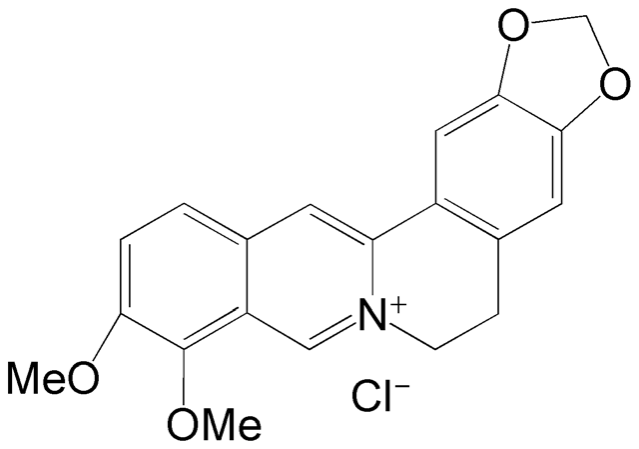
The structure of Berberine hydrochloride.

Berberine hydrochloride (BBH,Fig. 1), an alkaloid originally isolated from Huanglian (*Coptis chinensis*), has been extensively used in China as a nonprescription drug to treat diarrhea caused by bacteria since 1950s with a confirmed safety. In recent years, it has been demonstrated to possess a variety of pharmacological activities like antimicrobial[1], antineoplastic, antiviral, antiinflammatory[2], antiprotozoal, antidiarrheal, antileishmanial[3] ones, etc. The analytical methods for the determination of BBH were HPLC [4], [5], fluorescence quenching and enhancement methods [6], [7], [8], resonance Rayleigh scattering[9], visual colorimetric method[10] The spectrofluorimetric method has been widely used in the determination of biological samples environmental substances and pharmaceutical since it is highly sensitive, selective, easily operated and economic.

**Figure 2:**
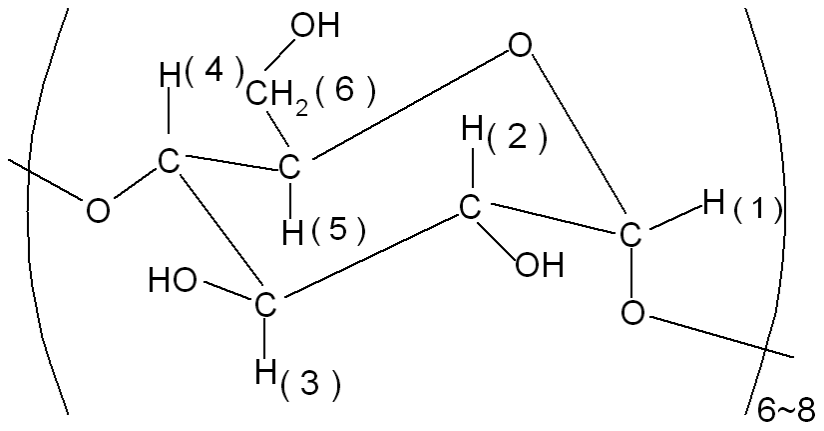
The structure of CD oligosaccharide.

Cyclodextrins (CDs, Fig.2) are cyclic oligosaccharides consisting of (α-1,4)-linked α-d-glucopyranose units, which have the property of forming inclusion complexes with guest molecules which possess suitable polarity and dimension. Having the ability to form inclusion complex by allowing other molecules into their hydrophobic cavity, CDs have been successfully used to improve solubility, chemical stability and bioavailability of a number of poorly soluble compounds.

In this work, we found that an obvious increase of fluorescent intensity was observed when β-CD was added to the aqueous solution of BBH. So, the study of the interaction between β-CD and BBH was carried out by spectrofluorimetry. The BBH/β-CD inclusion complex was prepared by freeze drying method and characterized by infra-red spectrograms. Based on the enhancement of the fluorescent intensity, BBH in aqueous solution was determined with high sensitivity and selectivity by spectrofluorimetry. The proposed method was applied to the determination of BBH in tablets with satisfactory results. Compared with HPLC and visual colorimetric method, the proposed method is fairly accurate, rapid, reproducible and has high sensitivity and wide linear range.

## Experimental

### 2.1Apparatus

All fluorescent measurements were measured with an RF-5301spectrofluorimeter (Shimadzu, Kyoto, Japan), equipped with a xenon lamp and1.0 cm quartz cells. Infra-red spectra were obtained from a Nicolet Nexus 670 FT-IR spectrophotometer (Madison, USA), equipped with a germanium attenuated total reflection (ATR) accessory, a DTGS KBr detector and a KBr beam splitter. Buffer solutions were made with a pHS-3C digital pH-meter (Shanghai Lei Ci Device Works) with a combined glass-calomel electrode. The sample chamber accommodated a thermostated cuvette holder, controlled to 25±1°C via a CS-50 constant temperature circulator (Chongqing Experimental Equipment Works, Chongqing, China).

### 2.2 Reagents

Berberine hydrochloride (99.7%) (purchased from National Institutes of Food and Drug Control, Beijing, Chian) was used as received without further purification. Its stock solution (0.10 mg/mL) was prepared with ethanol. β-CD was purified by recrystallization in double-distilled water, followed by vacuum drying at ca.60°C for 12 hours. 1.00×10^−2^ mol/ L β-CD was used. 0.10 mol/L of Tris-HCl buffer solution (pH = 9.00) was prepared. Other chemicals used were of analytical reagent grade or higher grade. Double-distilled water was used throughout.

### 2.3 Synthesis of β-CD-Berberine hydrochloride inclusion complex

0.8 g of BBH was dissolved in 50 ml β-CD saturated solution at 60°C with vigorously stirring. Then 5 ml ethanol was added. The mixture solution was stirred at 60°C until the yellow precipitate appeared. After the mixture was cooled to room temperature, it was stirred for 8 h at ambient temperature. The reaction mixture was allowed to store overnight at 4°C, then centrifugalized and filtered on a sintered glass filter. The product was obtained and washed sequentially with ethanol then dried in a vacuum oven at an elevated temperature (50∼55°C).

### 2.4 Experimental procedure

#### 2.4.1Calibration graph

Different aliquots of the BBH stock solution, 2.00 mL of 1.00×10^−2^ mol/L β-CD and 2.00 mL of 0.10 mol/L Trsi-HCl buffer solution (pH = 9.00) were added into a series of 10 mL colorimetric tube sequentially. The mixture was diluted to the mark with doubly distilled water, shaken thoroughly and equilibrated at 25±1°Cfor 15 min. Then the fluorescent intensity of the solution was measured at 431 nm/540 nm against a reagent blank.

#### 2.4.2 Determination of the apparent formation constant

Into a series of 10 mL colorimetric tubes were added 0.05 mL of 0.10 mg/mL BBH, varied amounts of 0.01 mol/L β-CD and 2.00 mL of 0.10 mol/L Tris-HCl buffer solution (pH = 9.00). The mixed solution was diluted to 10 mL with doubly distilled water, shaken thoroughly and equilibrated at 25±1°Cfor 15 min. Then the fluorescent intensity of the solution was measured at 431 nm/540 nm against a reagent blank.

#### 2.4.3 FT-IR spectra

FT-IR measurements were carried out at room temperature on a Nicolet Nexus 670 FT-IR spectrometer (America) equipped with a Germanium attenuated total reflection (ATR) accessory, a DTGS KBr detector and a KBr beam splitter. All spectra were taken via the Attenuated Total Reflection (ATR) method with resolution of 4 cm^−1^ and 60 scans.

## Results and Discussion

### 3.1 Fluorescence Spectra

**Figure 3:**
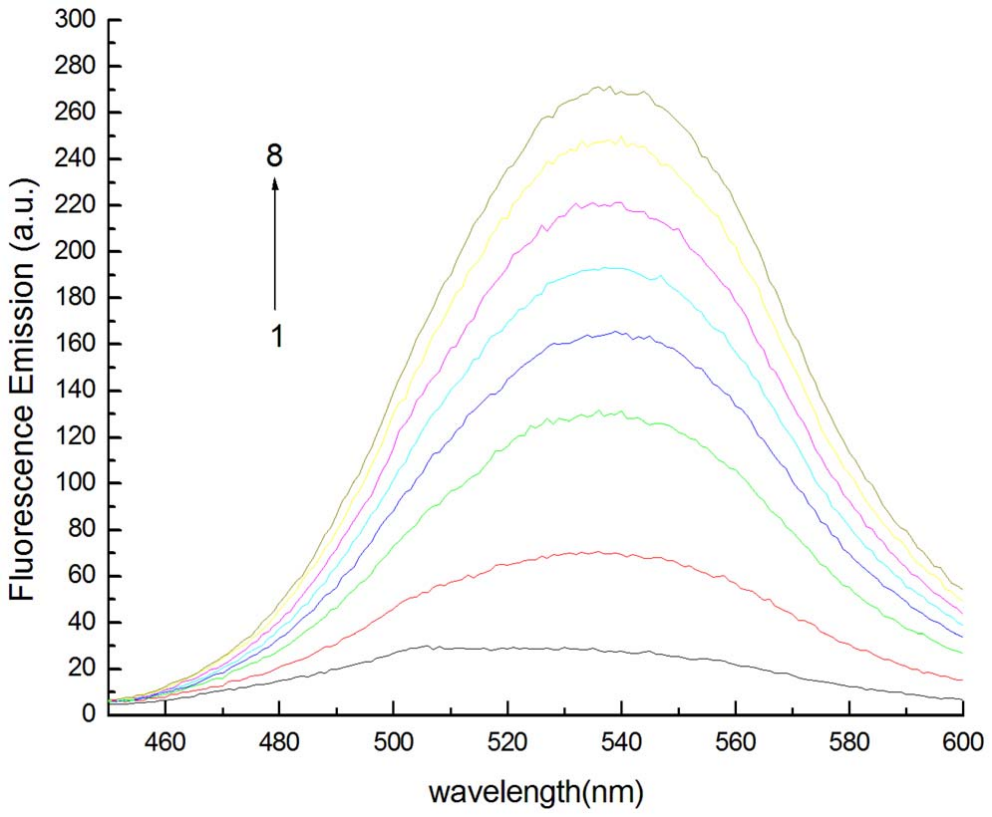
Fluorescence emission spectra. C_β-CD_: from top to bottom(1–8):0.0, 2.0,4.0,6.0,8.0,10.0,12.0, 14×10^−4^ mol/L. pH = 9.00, C_berberine hydrochloride_ = 2.00 µg/mL.

The fluorescence spectrum of BBH was measured with and without β-CD. As can be seen in Fig. 3, a dramatic increase in fluorescent intensity is observed when β-CD is added to the aqueous solution of BBH and the fluorescent signals are intensified with increasing concentration of β-CD. And the maximum emission wavelength has red shifted.

To demonstrate that the spectral changes are not due to a solvent effect caused by high concentration of β-CD, we tested the effect of glucose on the spectrum of BBH. The addition of glucose to a 2.00 µg/mL of BBH aqueous solution caused neither spectral shifts nor intensity changes in emission spetrum of BBH. Therefore, we confirmed that the β-CD-induced changes of the fluorescent spectra of BBH indicated the formation of supramolecular complex between BBH and β-CD.

### 3.2 IR Characterization of β-CD-BBH inclusion complex

**Figure 4:**
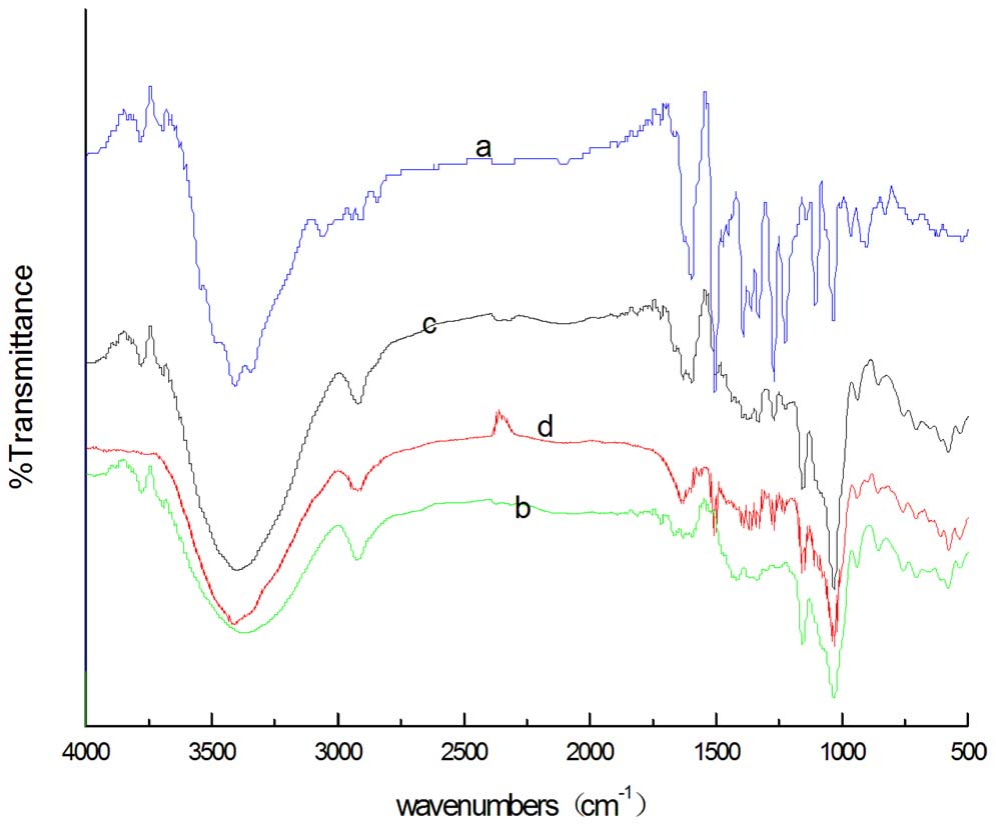
The infra-red spectragrams of BBH(a), β-CD(b), BBH/β-CD physical mixture(c), and the BBH/β-CD inclusion complex (d).

As we known, the characteristic IR absorption frenquency of CD covered the whole 400∼3800 cm^−1^ absorption band, so some characteristic IR absorption peaks of small organic guests in the spectra of the inclusion complex or the host/guest physical mixture might be covered up and was hard to be recognized. Meanwhile, some covered characteristic IR absorption peaks of the guest in the spectra of host/guest physical mixture could appear and red or blue shift in the spectra of the inclusion complex. Thus the variation of the shape, shift and intensity of the absorption peak of the guest or host could provide enough information for the occurrence of the inclusion[11]. By comparision of IR spectra of BBH (Fig.4a), β-CD(Fig.4b), the physical mixture of BBH and β-CD(Fig.4c) and the inclusion complex (Fig.4d), it could be seen that the spectrum of the physical mixture was essentially the combination of BBH and β-CD, which indicated that physical mixture cannot lead to inclusion; there were apparent differences between the spectra of the inclusion complex(Fig.4d) and physical mixture(Fig.4c) and some characteristic IR absorption peaks of BBH and β-CD changed obviously in inclusion complex: the absorption peaks of BBH at 3407.91 cm^−1^, 3055.81 cm^−1^, 2845.10 cm^−1^ disappeared,while there was a strong and wide absorption peak at 3415.45 cm^−1^; the absorption peaks within the scope of 3508-2914 cm ^− 1^ also disappear, at the same time, two new absorption peak at 2941.24 cm ^− 1^ and 2921. 96 cm ^− 1^ appeared; in addition, there were apparent differences within the scope of 1000–1260 cm ^− 1^, the absorption at 1031.19 cm^−1^ significantly enhanced. Based on these facts, it could be concluded preliminarily that BBH was included into the β-CD cavity to form a supramolecular inclusion complex.

### 3.3 Apparent association constant

The apparent association constant of the inclusion complex can be determined by the following method:

Assuming that the composition of the complex was 1∶1, the following expression can be written as: 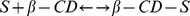



The formation constant of the complex (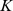) is given by: 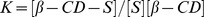



Where [β-CD], [S] and [β-CD-S] are equilibrium concentrations. An apparent constant association constant value for the inclusion complex can be determined through the typical double reciprocal plots [12].
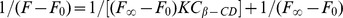



Where F is the fluorescent intensity of the BBH solution at each β-CD concentration tested, F_0_ and F_∞_ are the fluorescent intensity in the absence of β-CD and when all the BBH molecules are complexed, respectively. It was taken into account that (1) β-CD is in a large excess with respect to BBH and therefore its free and analytical concentration are similar; (2) the variations in the fluorescent signals are proportional to the complex concentration and (3) at high β-CD concentration essentially all of the BBH molecules are complexed.

**Figure 5:**
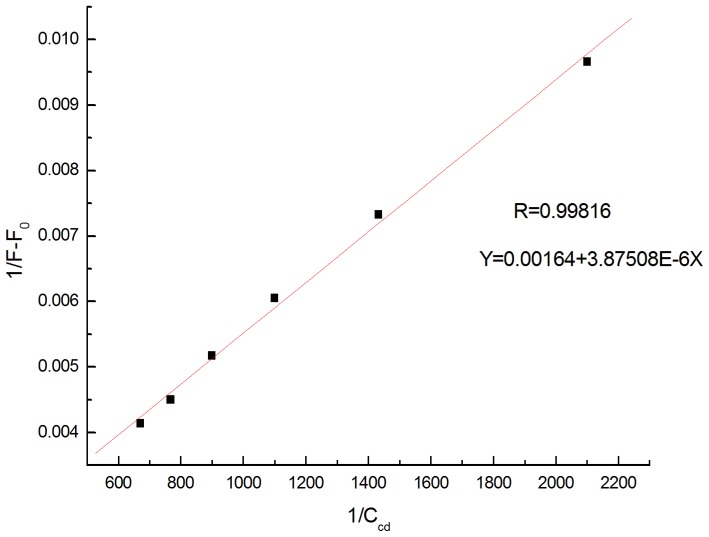
Plot of 1/(F−F_0_) versus 1/C_β-CD_.

The good linear relationship obtained when 1/ (F-F_0_) was plotted against 1/C_CD_ supported the existence of a 1∶1 complex (R = 0.99816, Fig.5). Its apparent association constant was determined to be 4.23×10^2^ L/mol.

**Figure 6:**
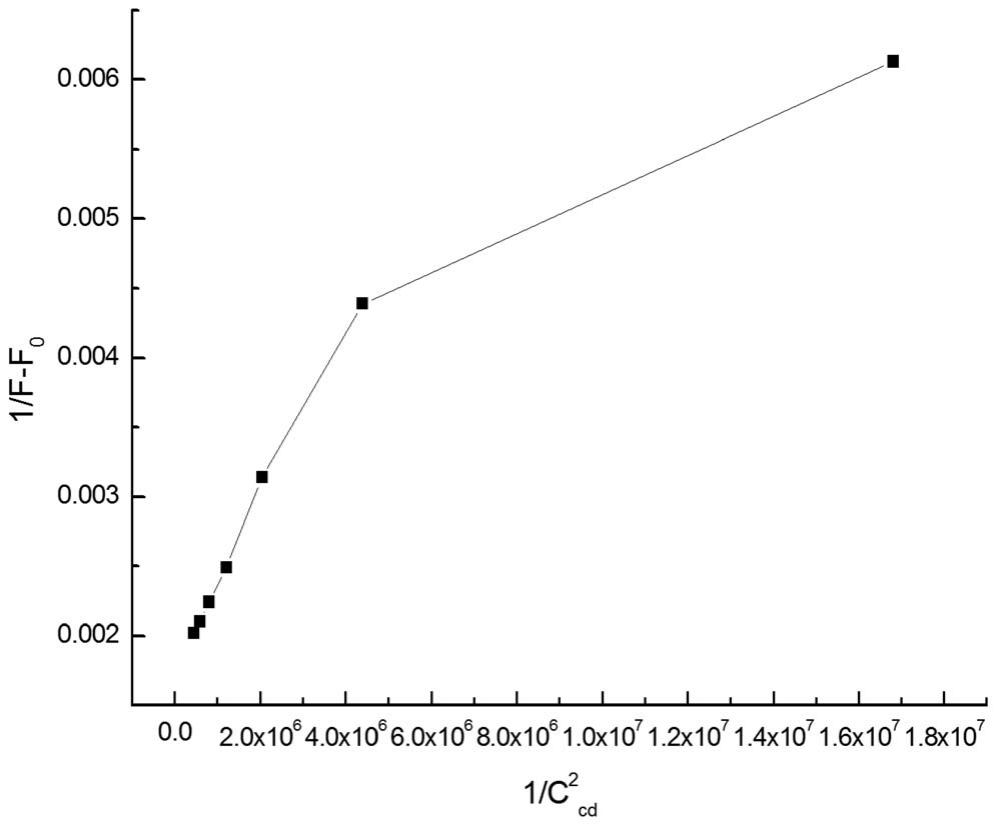
1/(F-F_0_)versus 1/C_β-CD_^2^.

Assuming that BBH and β-CD formed a 1∶2 complex, the following expression can be written as:
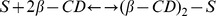



The formation constant of the complex (K’) is given by:
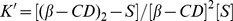



If 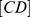
»[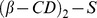]»[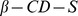], then the following expression is obtained:
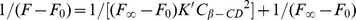



When making a plot of 1/(F-F_0_) against 1/[β-CD]^2^, no linear relationship can be observed (Fig. 6), which indicated that the composition of the complex wasn’t 1∶2.

### 3.4 Inclusion complex thermodynamics

**Figure 7:**
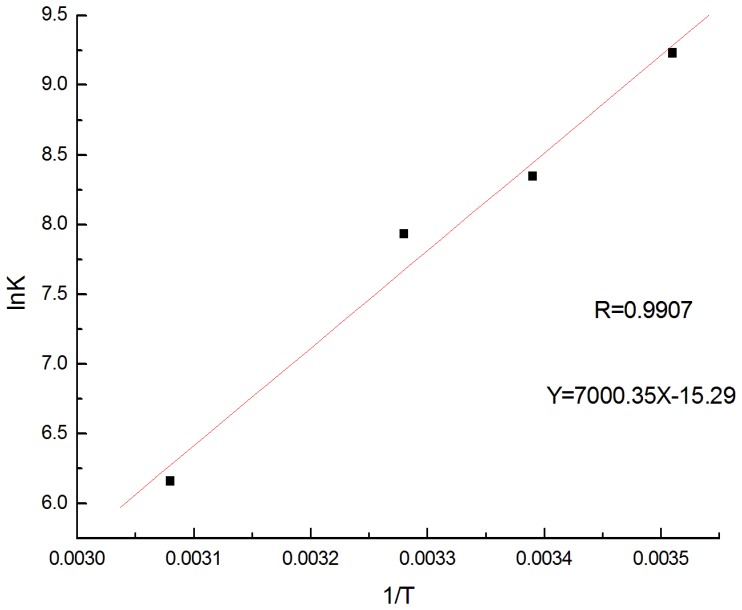
Plot of lnK versus 1/T.

The thermodynamic parameters, standard free energy (ΔG^0^), enthalpy (ΔH^0^) and entropy (ΔS^0^) for inclusion complex of BBH with β-CD were obtained from the Van’t Hoff equation: lnK = -ΔH^0^/RT+ΔS^0^/R (Fig. 7). The ΔH^0^ and ΔS^0^of the complex formation were calculated from the slope and intercept by plotting lnK vs. 1/T and ΔG^0^ was obtained according to the equation: ΔG^0^ = ΔH^0^-TΔS^0^. The results were shown in Table 1. As can be seen, when the temperature increases from 284.6K to 324.6K, the ΔH^0^ is negative, indicating that the complex dissociates when the temperature increases. The fact that ΔS^0^ is a small negative value may be attributed to the two reverse course: when BBH molecules are included into the cavities of β-CD, some water molecules are released, which leads to the increase of entropy. The net result of the two reverse courses is that the entropy of the system decreases slightly.

**Table 1:** Apparent formation constant (K), enthalpy (ΔH^0^), entropy (ΔS^0^), and standard free energy (ΔG^0^) changes of β-CD: berberine hydrochloride complex as a function of temperature.

	T (K)			
	284.6	294.6	304.6	324.6
lnK	9.23	8.35	7.93	6.16
△G^0^(kJ/mol)	−54.583	−54.456	−54.329	−54.075
△H^0^(kJ/mol)	−58.198			
△S^0^ (kJ/mol)	−0.0127			

### 3.5 The optimization of analysis conditions

#### 3.5.1 Influence of pH

**Figure 8:**
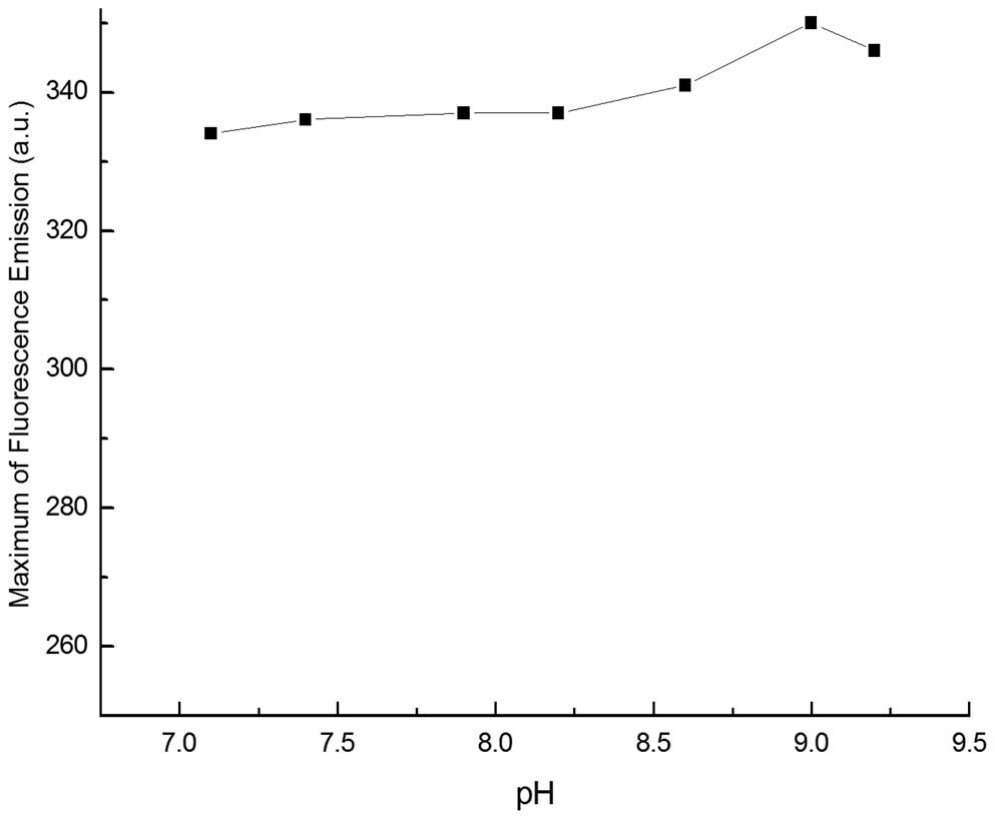
Influence of pH on the fluorescent intensity of the complex C = 2.00 µg/mL, C_ β-CD_ = 1.00×10^−3^ mol/L, λ_ex_/λ_em_  =  431/540 nm.

The pH dependence of the system was studied over the range of 7.00∼9.10. The results were shown in Fig. 8. As can be seen, the fluorescent intensity was relatively high and almost remained constant over the pH range of 8.70∼9.10. Therefore a pH of 9.00 was fixed with the use of Tris-HCl buffer solution.

The effect of the buffer will be lost as a result of a too small quantity of the buffer solution. While the buffer solution is excessive, the ionic strength will be too strong and then influence the fluorescence intensity. So the influence of the volume of the buffer solution was detected. As the volume of the buffer solution added (from 1.00 to 3.00 mL) had little effect on fluorescence intensity, 2.00 mL of buffer solution was used in subsequent experiments.

#### 3.5.2 Effect of the β-CD concentration

**Figure 9:**
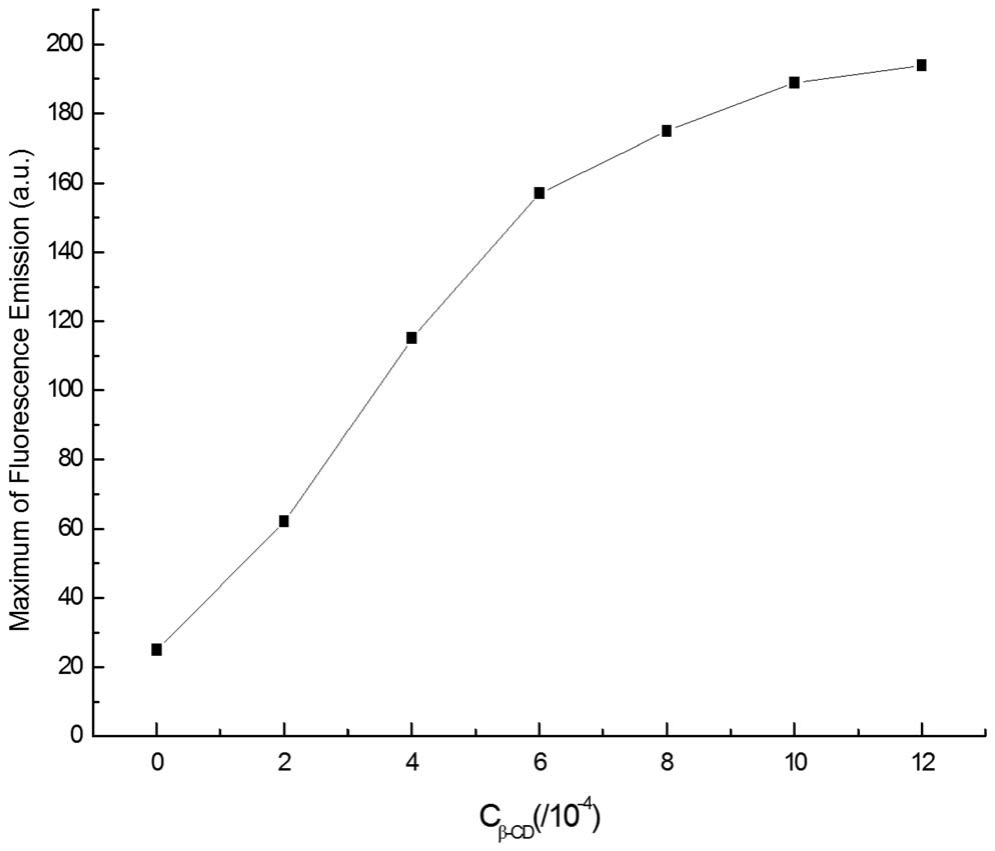
Effect of the β -CD concentration. C_berberine hydrochloride_ = 2.00 µg/mL, pH = 9.00.

Influence of β-CD concentration on the fluorescence intensity was shown in Fig. 9. As can be seen, with increasing concentration of β-CD the fluorescence intensity of the complex also increased. Meanwhile the fluorescence intensity increased slowly when β-CD concentration was above 1.00×10^−3^ mol/ L. Thus, 1.00×10^−3^ mol/ L β-CD was used.

### 3.6 Analytical characteristics

Under the optimum experimental conditions, there was a linear relationship between the fluorescence intensity and the concentration of BBH in the range of 1.00∼4.00 µg/mL. The regression equation was F = 119C(µg/mL)- 4.167, with a correlation coefficient of 0.9966. The detection limit, as defined by IUPAC [13], was determined to be 5.54 ng/mL according to formula of *C* =  *KS*_0_/*S*, where the value of *K* was taken as 3, the standard deviation was 0.22 obtained from a series of 11 reagent blanks, and *S* was the slope of the standard curve. The relative standard deviation (R.S.D.) was 0.95% obtained from a series of 11 standards each containing 2.00 µg/mL of BBH.

Based on the remarkable increase of the fluorescent intensity of BBH produced through the complex formation, a spectrofluorimetric method for the determination of BBH in bulk aqueous solution in the presence of β -CD was developed. For the purpose of comparison, calibration graph of BBH in the absence of β -CD was also constructed. The analytical characteristics obtained are shown in Table 2.

**Table 2:** Analytical characteristics of the method.

Analytical characteristics	The proposed method	In the absence of β -CD
Linear regression equation^a^	F = 119C(µg/mL)- 4.167	F = 13.65C(µg/mL)- 2.372
Linear range(µg ml^−1^)	1.0∼4.0	3.0∼12.0
Correlation coefficient	0.9966	0.9982
S_0_ b	0.22	0.34
LOD^c^ (ng/mL)	5.44	74.7
LOQ^d^ (ng/mL)	18.4	246.5
RSD^e^(%)	0.95	1.6

a The number of data for each calibration graph correspond to 8 different concentration levels, with three replicates for each level.

b The standard deviation obtained from a series of 11 blanks solutions.

c Limit of detection calculated according to IUPAC definitions: 3S^0^/K, where K is the slope of the calibration graph.

d Limit of quantification calculated according to the IUPAC definition: 10S_0_/K.

e Relative standard deviation obtained from a series of 11standards each containing 0.50 µg ml^−1^berberine hydrochloride.

As can be seen, the analytical characteristics in the presence of β–CD were significantly improved. Obviously decreases in both the limit of the detection (LOD) and the limit of quantification (LOQ) were achieved with respect to the solutions without β –CD. So, it can be concluded that an improved sensitively spectrofluorimetric determination of BBH in the bulk aqueous solution can be achieved in the presense of β –CD.

### 3.7 Influence of usual excipients

Before the proposed method was applied to real samples, the influence of the commonly used tablet excipients on the determination of 2.00 µg/mL BBH was studied. A 2000-fold mass excess of each excipient over BBH was tested first. If interference occurred, the ratio was reduced progressively until the interference ceased. The criterion for interference was fixed at a ±5% variation of the average fluorescent intensity calculated for the established level of BBH. The results (Table 3) showed that the determination was free from the interference of the usual excipients.

**Table 3:** Influence of excipients normally used in the tablets formulation (tolerance error ± 5.0%).

Tolerance ratio in mass	Excipient
50003000	K^+^, Na^+^, Ca^2+^, Mg^2+^sorbitol gelatin
2500	Cl^-^, lactose, starch
2000	mannitol, gum acacia power,
1500	sodium carboxymethy-celluose, sodium acetate
1000	sucrose, glucose, boracic acid, Fe^3+^
500	merhyl cellulose, Polyethylene glycol 4000

## Determination of BBH in tablets

BBH content in tablets were determined following the proposed spectrofluorimetric method. The results are shown in Table 4. As can be seen, results obtained by the proposed method agree well with the label claim.

**Table 4:** Determination of berberine hydrochloride in tablets (n = 5, p = 95%).

Pharmaceutical preparations	Label claim(mg/tablet)	The proposed method(mg/tablet)	RSD
fu fang huang lian su tablets	30	30.06±0.32	0.86
berberine hydrochloride	100	98.76±0.12	0.21

To evaluate the extraction procedures and the accuracy of the method, a recovery assay was performed. The recoveries of BBH added to different concentration sample solutions were shown in Table 5. It can be seen that the recoveries were 99.5%∼103.2% and did not beyond the recommended limits of within ±5% of the indicated amount.

**Table 5:** The recoveries of the determination of berberine hydrochloride in tablets.

Sample number	Sample content (µg/mL)	Berberine hydrochloride added (µg/mL)	Berberine hydrochloride found (µg/mL)	Recovery (%)
1	0.4978	0.5000	0.9954	99.5
2	0.9956	1.0000	2.0243	102.8
3	1.4934	1.5000	3.0421	103.2

## Conclusions

In this study, the interaction between β-CD and BBH was carried out by spectrofluorimetry and infra-red spectrograms. Because of the enhancement of the fluorescent intensity of BBH, a sepctrofluorimetric method for the determination of BBH in bulk aqueous solution in the presence of β-CD was developed. The linear range was 1.00∼4.00 µg/mL with the detection limit of 5.54 ng/mL. The proposed method was successfully applied to the determination of BBH in tablets.
